# Bacterial communities and soil functionality in artificially remediated vegetation of the three gorges reservoir zone

**DOI:** 10.3389/fpls.2025.1550306

**Published:** 2025-04-28

**Authors:** Farkhanada Naz, Muhammad Arif, Tan Xue, Yangyi Chen, Shahid Ullah Khan, Li Changxiao

**Affiliations:** ^1^ Key Laboratory of Eco-Environments in the Three Gorges Reservoir Region (Ministry of Education), College of Life Sciences, Southwest University, Chongqing, China; ^2^ Biological Science Research Center, Academy for Advanced Interdisciplinary Studies, Southwest University, Chongqing, China; ^3^ School of Tourism Ecology and Environment, Guilin Tourism University, Guilin, China; ^4^ Integrative Science Center of Germplasm Creation in Western China (Chongqing) Science City and Southwest University, College of Agronomy and Biotechnology, Southwest University, Chongqing, China

**Keywords:** artificial remediation plant, riparian ecosystem, soil microbial diversity, KEGG pathways, PICRUSt analysis

## Abstract

Riparian zones maintain biodiversity, cyclic nutrients, and regulate water quality. However, their stability is increasingly threatened by human activities such as dam construction and climate variability. This study focuses on the riparian zones of the Three Gorges Dam Reservoir (TGDR), a region marked by fluctuating water levels and a subtropical southeast monsoon climate. We investigated the seasonal and vegetation-specific dynamics of soil properties and microbial communities in riparian zones dominated by artificially remediated plants (ARPs) in the TGDR. The selected ARP species included the herbaceous *Cynodon dactylon* (CD) and *Hemarthria altissima* (HA), known for their capacity for rapid soil stabilization, and the tree species *Salix matsudana* (SM) and *Taxodium distichum* (TD), which enhance nutrient cycling through litter inputs and root exudates. These species were evaluated across spring (T1), summer (T2), and autumn (T3). Our analysis of 360 soil samples led to the generation of high-quality sequences that provided insights into microbial diversity. Principal component analysis identified organic matter, ammonium nitrogen, and total nitrogen as the main contributors to soil property variance, explaining 53.68% in T1, 51.52% in T2, and 56.37% in T3 of the variance (p < 0.01). Correlation analysis highlighted a positive relationship between soil pH and *Nitrospirae* (r = 0.603) and *Proteobacteria* (r = 0.558). Enzyme activity varied by season, with acid phosphatase activity peaking in T3 and invertase activity highest in T1. This study also made functional predictions and identified pathways pertinent to metabolism, genetic information processing, and environmental signal transduction. There were seasonal shifts in metabolic pathways, such as an increase in carbohydrate metabolism in T3 via TD. In addition, there was a rise in amino acid metabolism in T3 via CD. Our assessment of microbial diversity uncovered 68 bacterial phyla, with *Proteobacteria* and *Acidobacteria* emerging as the dominant taxa. The results indicate that ARPs positively influence microbial health, nutrient cycling, and overall ecosystem integrity. These findings hold significant implications for riparian ecosystem restoration in regions experiencing environmental changes.

## 1 Introduction

Riparian zones serve as the critical interface between land and water, playing an essential role in global ecosystem stability (Zhang X. et al., 2020). At the heart of these functions lies the biological fertility of the soil. This fertility is supported by diverse microbial communities that facilitate nutrient cycling, carbon sequestration, and overall ecosystem health. The unique conditions of hydraulic systems, soil composition, and vegetation characteristics shape these zones (Gong et al., 2023). Biological life within riparian zones contributes significantly to habitat support, riverbank stabilization, impurity filtration, and nutrient recycling (Yang et al., 2023). In addition, these areas offer vital functions for the accommodation and protection of various species, including those that are threatened, while also helping to regulate water quality (Yu et al., 2023). However, anthropogenic disturbances such as flooding and dam construction have adversely affected these ecosystems (Zhao et al., 2020). The riparian zones of the Three Gorges Dam Reservoir (TGDR) exemplify the consequences of such disturbances, showing erratic water levels induced by dam modifications (Li et al., 2022a). These human-induced alterations have rendered riparian ecosystems vulnerable, leading to severe impacts such as soil erosion, nutrient depletion, and vegetation loss (Yuan et al., 2023; Li et al., 2021a). These disturbances disrupt hydrological processes but also compromise soil structure and diminish microbial functions essential for maintaining soil fertility. Consequently, these changes have degraded vital life-sustaining functions, including carbon sequestration, nutrient cycling, and species protection (Peralta et al., 2013). To address these challenges, adaptive restoration approaches are necessary to effectively manage hydrology and mitigate climate change impacts in these vulnerable environments.

The use of an artificially remediated plants (ARPs) strategy offers a promising solution for restoring ecosystem functions (Naz et al., 2024a; Zhang X. et al., 2020). Effective ARPs possess key characteristics, including deep root systems, high flood tolerance, and rapid regrowth. These traits stabilize soil but also promote beneficial interactions between plants and microbes. As a result, they help reduce soil erosion, improve nutrient storage, and enhance the symbiotic relationships among plants, soil, and microbes (Adamczyk et al., 2021; Liu et al., 2023). For example, *Cynodon dactylon* (CD) and *Hemarthria altissima* (HA) are two herbaceous plants known for their robust root systems, which can quickly stabilize soil in areas threatened by erosion (Iori et al., 2019). Tree species, particularly *Salix matsudana* (SM), play a crucial role in enhancing nutrient cycling and organic matter accumulation. *Taxodium distichum* (TD) also helps with this process through its root exudates and litter decomposition, both of which increase the variety of microbes (Shiva Khanal et al., 2024). Ultimately, a promising approach to overcoming degraded riparian zone challenges involves utilizing ARPs that consist of plant species with specialized traits aimed at stabilizing soil, enhancing microbial diversity, and reestablishing critical ecosystem function.

Maintaining the health and stability of natural environments depends on the performance of critical ecosystem functions. Although ARPs show immense potential for use in dam-regulated systems like the TGDR, their application in these settings faces significant challenges. The subtropical southeast monsoon climate of the TGDR leads to substantial fluctuations in temperature, rainfall, and water levels throughout the year. These variations profoundly affect soil properties, plant growth, and microbial community dynamics (Cao et al., 2021; Gao et al., 2023). During wet seasons, increased soil moisture and nutrient availability enhance microbial activity, while drier seasons can disrupt microbial community composition (Wang et al., 2023; Zhong et al., 2018). To effectively restore these ecosystems year-round, ARP strategies must adapt to dynamic environmental conditions. Despite their promising characteristics, little is known about how seasonal variations and species-specific traits influence soil microbial dynamics in dam-regulated riparian zones. This knowledge gap must be addressed to develop targeted restoration strategies. To advance our understanding, it is crucial to analyze soil bacterial dynamics across different seasons and vegetation types. Such analysis will help evaluate species turnover and factors influencing microbial community structures. By integrating insights into soil dynamics—encompassing physical, chemical, and enzymatic properties—we can gain a clearer understanding of how these characteristics vary under different conditions across plant types. This comprehensive approach not only enhances our understanding of soil health but also characterizes the interactions between plant species and microbial communities. These interactions are essential for successful riparian restoration.

Ecosystems rely on microbial communities to facilitate nutrient movement and decompose organic matter (Zhai et al., 2024). These communities thrive in riparian environments, which are crucial for ecosystem health. The need exists to study microbial dynamics to identify key taxa that drive soil health, with the understanding that successful ARPs depend on promoting beneficial microbial communities (Naz et al., 2024b). Utilizing next-generation sequencing and bioinformatics tools, such as PICRUSt2 and KEGG Orthology, researchers can uncover microbial pathways that reduce sulfate, fix nitrogen, and cycle phosphorus (Li et al., 2021b; Singh et al., 2022). Analyzing these systems can enhance our understanding of how microbial communities mediate essential ecosystem functions and whether they can be manipulated to accelerate the restoration of degraded riparian zones. To address these knowledge gaps, this study employs a multi-season, multi-species approach to evaluate the effects of ARPs on soil properties, microbial diversity, and ecosystem function within the riparian zones of the TGDR. Specifically, the research focuses on three key questions: (1) How do ARPs influence soil properties, microbial diversity, and ecosystem functions in riparian zones? (2) How do seasonal changes affect ARP effectiveness in enhancing soil stability and microbial community health? (3) What are the most effective plant species (CD, HA, SM, and TD) for seasonally adaptive ARP applications?

Therefore, this study aims to fill gaps in knowledge surrounding ARP seasonal and vegetation-specific dynamics in riparian ecosystems. By combining information about the physical and chemical properties of the soil, the variety of microbes that live in it, and their functional pathways, this study will help create flexible, long-lasting restoration plans that make ecosystems more resilient in places like the TGDR that are controlled by dams. The findings could provide valuable insights into optimizing ARPs for both the TGDR and similar ecosystems worldwide, thus advancing the field of climate-resilient restoration strategies for riparian zones. This study endeavors to bridge critical knowledge gaps and provide actionable solutions to enhance restoration efforts in riparian ecosystems globally.

## 2 Materials and methods

### 2.1 Study area

The riparian zones of the Ruxi River basin, in Chongqing City, China (107°32′-108°14′E; 30°03′-30°35’N), serve as the study area (**Supplementary Figure 1**). It experiences a subtropical southeast monsoon climate with an annual mean temperature of 18.2°C, about 80% humidity, and annual rainfall of 1200 mm (Arif et al., 2022). The area boasts purple soil, a highly silty, highly erosion-prone lime-based soil (Tao et al., 2020). This region features purple soil characterized by a calcareous and highly silty composition, along with minimal clay fractions and organic matter. Its richness in iron oxide, combined with a silty texture and low clay and organic matter content, puts the soil at elevated risk of erosion. These soil attributes are crucial, as they influence the ecosystem’s ability to retain nutrients, establish microbial community structures, and ultimately affect the overall functionality of the zone (Peng and Yu, 2023). Seasonal flooding associated with the TGDR significantly influences soil and vegetation dynamics. We studied four vegetation types: herbaceous species (CD, HA) and tree species (SM, TD). These species were selected because of their ecological significance and adaptability to flood-prone riparian zone traits.

### 2.2 Species selection and justification

We strategically chose the vegetation types of CD, HA, SM, and TD based on their ecological significance, adaptive traits, and their role in riparian ecosystem restoration. CD is renowned for its rapid colonization ability in disturbed habitats, primarily due to its extensive network of fibrous roots and underground rhizomes. These characteristics stabilize the soil quickly and facilitate early succession processes (Kotangale et al., 2025). Species of CD and HA are robust herbaceous species that have a large root system that stabilizes the soil and prevents erosion, particularly in seasonal flood-prone areas (Ye et al., 2020; Zeng et al., 2023). Similarly, the dense growth patterns of HA result in stands that effectively maintain soil moisture and provide protection against both waterlogging and drought, thereby offering a reliable habitat for various microbial communities (Neto et al., 2024). These grasses are indeed highly resistant to waterlogging and drought conditions and therefore are suitable for the hydrological regime of the TGDR (Wang et al., 2022; Zhang et al., 2021). However, flood-tolerant TD plays a key role in nutrient cycling and organic matter retention during wet seasons (Xia et al., 2018; Yun et al., 2023). Additionally, TD exhibits exceptional flood adaptation through its deep roots, which secure soil structure and sustain nutrient processes during severe water events. The functional traits exhibited by these species enhance the potential for rehabilitating disturbed riparian ecosystems (Guo et al., 2024). SM also grows in wet soil near waterways, which help a variety of microbes by providing root exudates and encouraging more complex interactions between plants, microbes, and soil (Yuancai et al., 2022). SM offers distinct benefits for riparian restoration, as this species contributes abundant leaf litter to the ecosystem while establishing complex below-ground microbial networks that drive nutrient cycling processes (Yang et al., 2022). These species make up a wide range of herbaceous and woody plants, which makes it possible to study how different types of plants affect soil physical and chemical properties, as well as the activities of enzymes, the movement of microbes, and the cycling of nutrients. This selection also aligns with the goals of this study to assess ecosystem functionality and resilience in TGDR riparian zones. Each species employs unique strategies adapted to various factors influencing soil stability, nutrient cycling functions, and the development of microbial communities across the variable environments of the TGDR (**Supplementary Figure 2**).

### 2.3 Experimental design

In the spring 2012 season, field investigation was conducted in the riparian zone of the Ruxi River Basin. It was aimed at Shibao Town in Zhongxian County. This experiment focused on four plant species, including CD, HA, TD, and SM. Despite eight consecutive years of flooding, the plants transitioned into a stable growth phase. The research project employed a detailed sampling approach across three key time points: The harvest season was early spring (May 2023, T1), summer (July 2023, T2), and autumn (September 2023, T3). To sample seasonal and vegetation-specific variability in soil properties, each season marked a critical growth stage for the plants. In order to represent soil characteristics across seasonal transitions, the study used a stratified sampling design. Three 1 m × 1 m quadrats were constructed within each vegetation type. There was at least 10 m between quadrats to allow for representative sampling within each vegetation type. We collected composite soil samples from the top 20 cm layer in each quadrat, an ecologically active zone for microbial and nutrient processes (Callaghan et al., 2022), along an S-shaped transect within each quadrat. In total, 360 composite soil samples were collected over three seasons, with 90 samples per vegetation type. The most significant impact of this design was that it allowed a flawless comparison of soil physical, chemical, and microbial properties under different environmental and vegetation conditions.

### 2.4 Data collection

#### 2.4.1 Physical, chemical, and enzymatic properties

Physical soil properties such as soil temperature (ST), soil moisture content (SMC), bulk density (BD), and oxidation-reduction potential (ORP) were measured using the standard gravimetric and potentiometric methods described by Chen et al. (2019). An elemental analyzer determined the total carbon (TC), total nitrogen (TN), and organic matter (OM) content while the soil pH was determined using electrode potentimetry (Muneer et al., 2022). The contents of total phosphorus (TP), available phosphorus (AP), and total potassium (TK) were measured by inductively coupled plasma emission spectroscopy (ICP-OES) and molybdenum-antimony colorimetry (Shi et al., 2022). Soil nitrate nitrogen (NO₃-N) and ammonium nitrogen (NH₄⁺-N) concentrations were measured by an automated colorimetric method (Arar and Collins, 1997).

Soil nutrient cycling functionality was assessed by measuring the activity of three key enzymes: The three cultivars were subjected to acid phosphatase (ACP), urease (URE), and invertase (INV) (Yin et al., 2018). The ACP activity was determined by the phenyl phosphate released to the incubated medium, whereas the URE activity was measured by the ammonia contained in the incubation medium (Ren et al., 2018). The quantification of INV activity was related to the amount of glucose produced during the incubation period. The enzymatic assays of these enzymes are crucial for the elucidation of biochemical pathways regulating nutrient dynamics in soil and are also effective indicators of microbial health (Litvinovich et al., 2020). All measurements were carried out in triplicate, as necessary for robust scientific reporting to guarantee accuracy and reliability of results.

#### 2.4.2 Microbial community analysis

The DNA of microbial communities was extracted according to standardized protocols with the DNeasy^®^ PowerSoil^®^ Kit (QIAGEN). Then, the integrity and concentration of the DNA were evaluated by using a NanoDrop spectrophotometer and agarose gel electrophoresis. Amplicon libraries were subsequently sequenced with high-throughput sequencing by using the Illumina NovaSeq platform, using the universal bacterial 16S V4-V5 region forward primer denoted as 515 F (5-GTGCCAGCMGCCGCGG-3) and the reverse primer as 907 R (5-CCGTCAATTCMTTTRAGTTT-3). The UPARSE algorithm (Liao et al., 2023) was used to cluster high-quality sequences at a 97% similarity threshold into operational taxonomic units (OTUs). The Ribosomal Database Project (RDP) database was used to classify the species by taxonomic classification. Species richness and evenness were evaluated with the alpha diversity indices of Chao1, Shannon, and Simpson, and compositional shifts in the beta diversity patterns were analyzed with the Principal Component Analysis (PCA), showing differences in seasons and vegetation types (Xu et al., 2023).

#### 2.4.3 Functional and pathway analysis

PICRUSt2 was used to conduct functional predictions, mapping OTU data to the KEGG Orthology (KO) pathways (Yang et al., 2023). Insights into microbial functional potential were gained on key metabolic functions of the studied organisms, such as amino acid and carbohydrate metabolism and nitrogen cycling. Seasonal and vegetation-specific dynamics in the abundances of microbial functional pathways were analyzed and demonstrated microbial adaptations and contributions to soil nutrient cycling.

#### 2.4.4 Statistical analysis

Seasonal and vegetation-specific variations in soil physicochemical properties, enzymatic activities, bacterial alpha diversity, and functional pathway abundances were evaluated by using one-way ANOVA. *Post hoc* multiple comparisons to see whether there were significant differences were carried out using Tukey’s Honest Significant Difference (HSD) test. PCA was used to assess relationships between soil parameters (e.g., pH, OM, and nutrient concentrations), microbial metrics, and to characterize seasonal and vegetation-specific patterns of soil properties and microbial community by identifying key drivers. The technique was applied to reduce data dimensionality while retaining the maximum variance, which enabled the visualization of complex interactions (Qiu et al., 2023). All the statistical analyses were done using SPSS 28.0, and graphical visualizations (bar plots and PCA scatter plots) were done using OriginPro 2023 and Excel 2021.

## 3 Results

### 3.1 Seasonal and vegetation-specific evaluation of soil bacterial sequencing data quality

**Figure 1:**
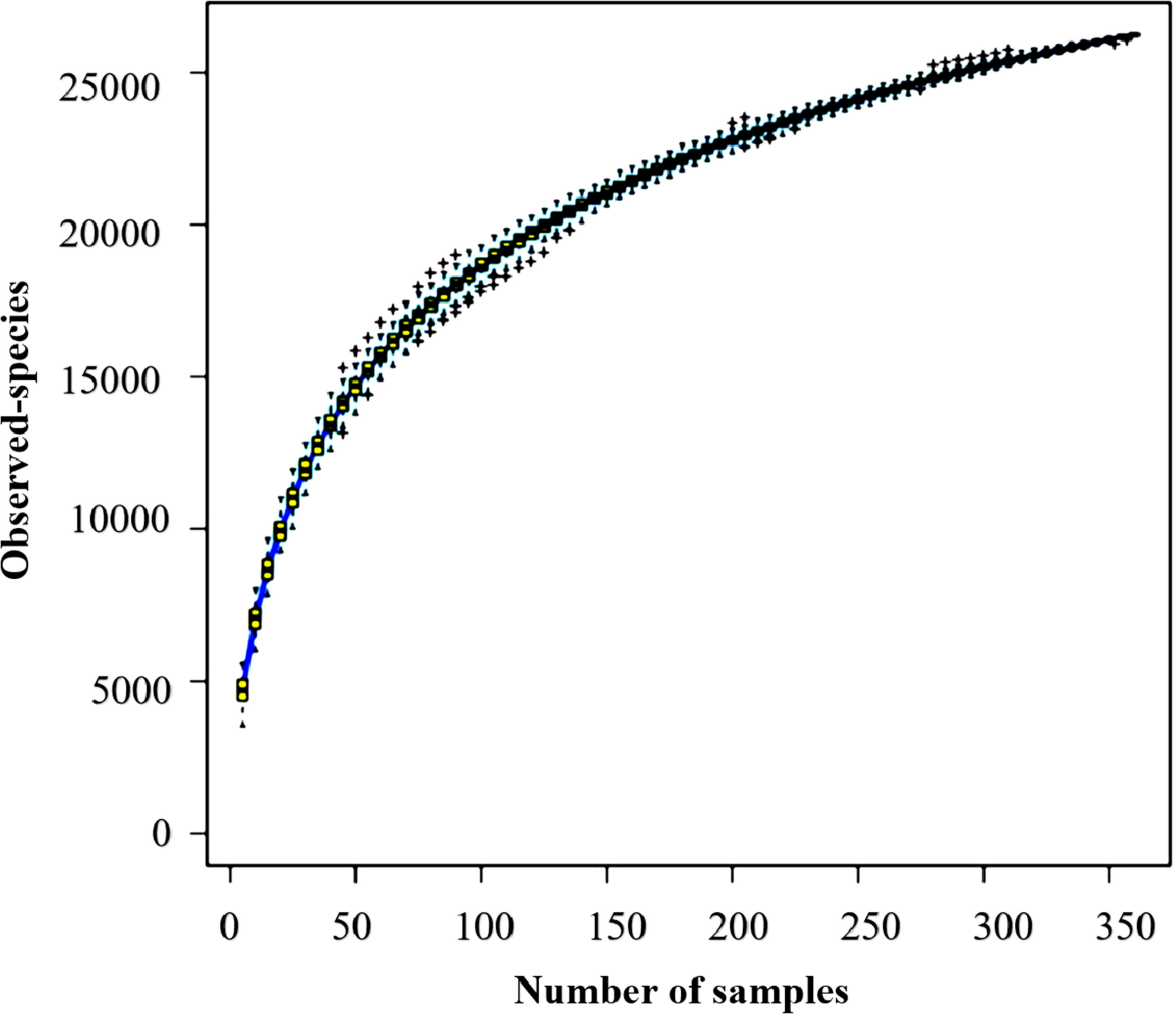
The species accumulation curve displays bacterial richness in 360 soil samples. The horizontal axis represents the number of sequenced samples, and the vertical axis represents the number of OTUs.

This study evaluated soil bacterial communities on 360 samples from three seasonal periods using a high-throughput sequencing approach. It is important to note that the value of 38,529,489 represents the total number of high-quality sequences obtained from all 360 samples, with each sample averaging 107,026 sequences (median = 102,483). Each sample contained between 22,979 and 280,162 high-quality sequences. After putting the sequences into OTUs and getting rid of the chimeric sequences, 34,221,897 sequences were left. These sequences ranged from 20,378 to 251,617 per sample, with 95,061 being the average and 89,816 being the median. We completed rarefaction to 20,378 sequences per sample to standardize analytical measures across samples. Species accumulation curve (**Figure 1**) revealed a rapid increase in bacterial richness with sample numbers from 1 to 100. This indicates the discovery of novel taxa during the initial sampling period. Once the sample size surpassed 100, the rate of species accumulation began to flatten, and after about 300 samples, the curve plateaued, indicative of very little incremental addition of new taxa. Therefore, this study illustrates that sequencing depth and sampling effort are sufficient to recover soil bacterial communities’ complete diversity. These results thus offer a firm basis for further downstream analyses, allowing us to characterize how soil microbial diversity relates to seasonal variations and vegetation types.

### 3.2 Seasonal and vegetation-specific soil dynamics: integrating physical, chemical, and enzymatic insights

#### 3.2.1 Physical properties

**Figure 2:**
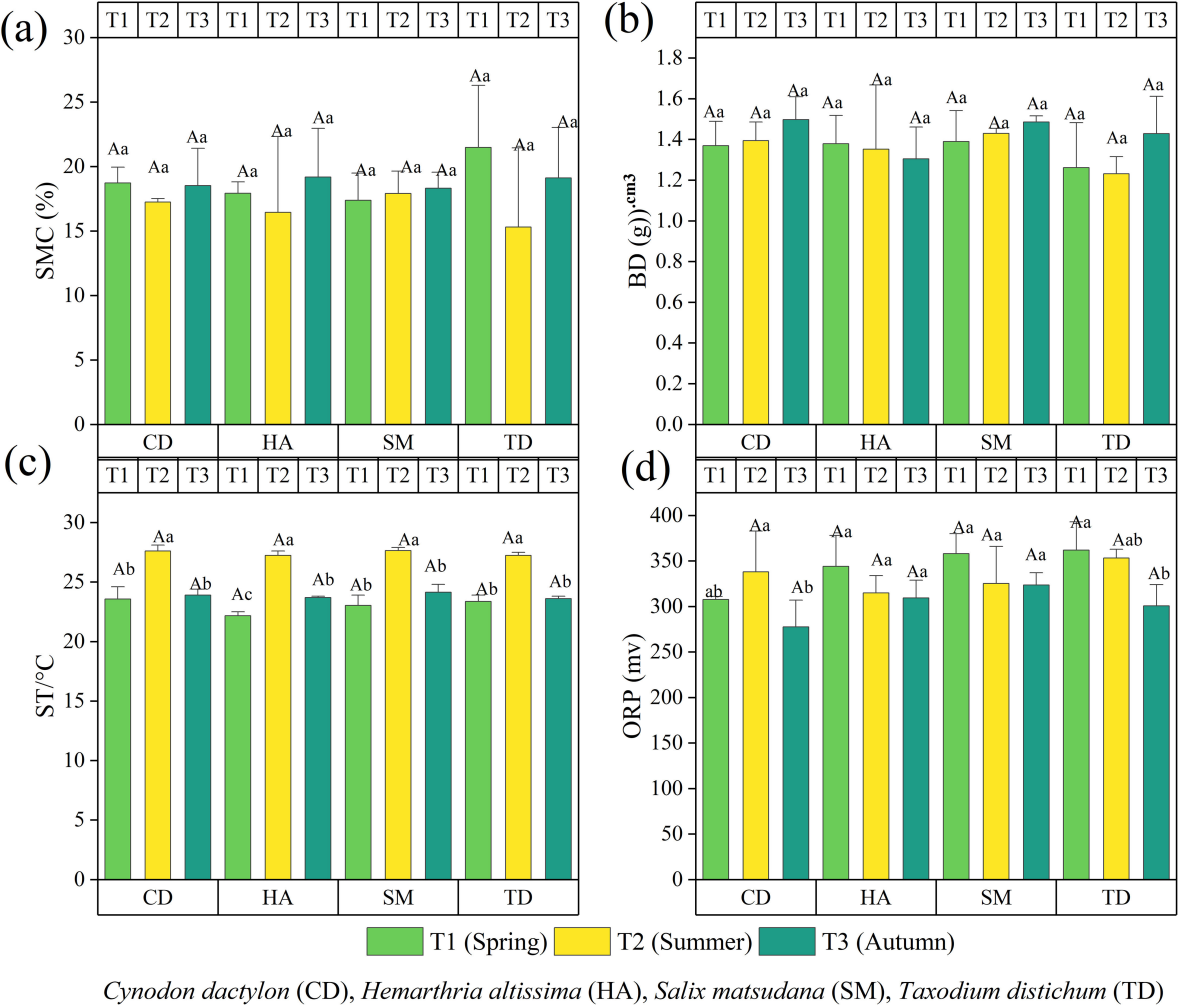
The bar graphs **(a–d)** illustrate the physical characteristics of the soil in the riparian zones of the Three Gorges Dam Reservoir (TGDR) in China across various timeframes. Abbreviations in the figure define soil moisture content, bulk density (BD), soil temperature (ST), and oxidation-reduction potential (ORP). Uppercase letters indicate significant differences between vegetation types in the same period, while lowercase letters show differences across periods (p < 0.05).

**Figure 2** illustrates the seasonal variation in soil physical properties in the riparian zones of the TGDR in China, assessed over three seasons (T1, T2, T3) for CD, HA, SM, and TD. SMC also showed seasonal variability with the highest SMC in T3 driven by rainfall, while TD peaked in T1, probably caused by spring precipitation. CD and HA herbaceous SMC were stable across seasons with a small increase in T3 (**Figure 2a**). TD had the highest BD in T3, but CD and HA were generally invariable with respect to seasonality (**Figure 2b**). ST inputs reached their maximum in T2 in all vegetation types (**Figure 2c**), and ORP peaked in T1 in TD consistent with dynamic redox regulation (**Figure 2d**). These findings emphasize the importance of seasonal precipitation, vegetation type, and organic matter dynamics on soil structure and redox balance.

#### 3.2.2 Chemical properties

**Figure 3:**
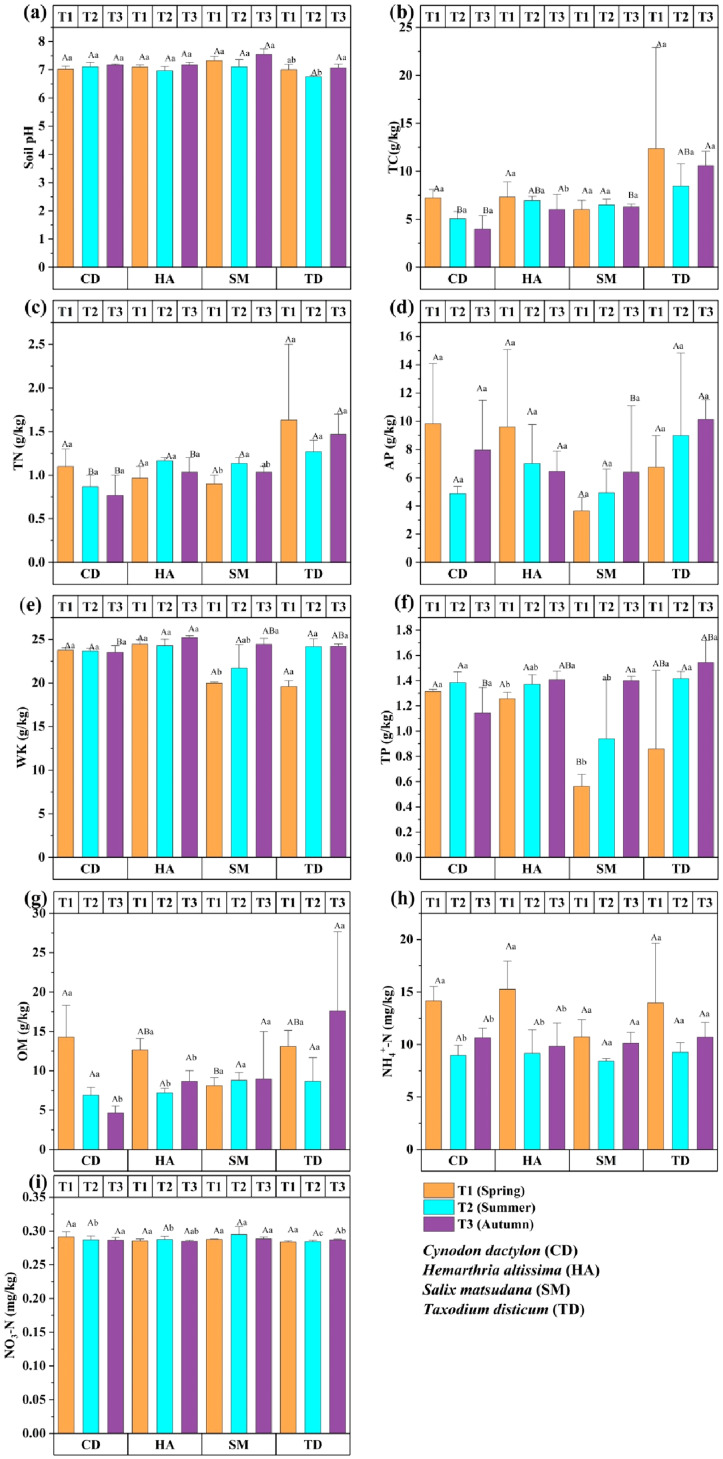
Bar graphs **(a–i)** show the chemical properties of four vegetations at different periods in the riparian zones of the TGDR in China. Abbreviations in figures defined as pH values (PH), total carbon (TC), total nitrogen (TN), available phosphorous (AP), whole potassium (WK), total phosphorous (TP), organic matter (OM), ammonium nitrogen (NH_4_^+^-N), and nitrate nitrogen (NO_3_-N). Uppercase letters indicate significant differences between vegetation types in the same period, while lowercase letters show differences across periods (*p < 0.05*).

**Figure 3** shows the seasonal variation in soil chemical properties in the riparian zones of the TGDR, assessed over three seasons (T1, T2, T3), highlighting significant seasonal fluctuations. Soil pH ranged from 6.5 to 7.5, lowest in T2 and highest in T3 for SM and TD, indicating enhanced buffering in T3 (**Figure 3a**). TC and TN peaked in T1 for TD and HA (*p < 0.05*), reflecting organic residue accumulation and nitrogen availability (**Figures 3b, c**). AP was highest in T3 for TD (*p < 0.05*), while HA showed the highest AP in T1 (**Figure 3d**). WK was stable for CD and HA, spiked in T2 for TD (*p < 0.05*), and declined in T2 for SM (**Figure 3e**). TP was highest in T3 for TD (*p < 0.05*), while HA showed moderate increases across seasons (**Figure 3f**). OM peaked in T3 for TD (*p < 0.05*) due to litterfall (**Figure 3g**). NH₄⁺-N peaked in T1 for CD and TD (*p < 0.05*), while NO₃⁻-N was highest in T1 for CD (*p < 0.05*), declining in later seasons (**Figures 3h, i**). These significant results underscore the influence of seasonal and vegetation types on nutrient cycling and soil fertility.

#### 3.2.3 Enzymatic activity

**Figure 4:**
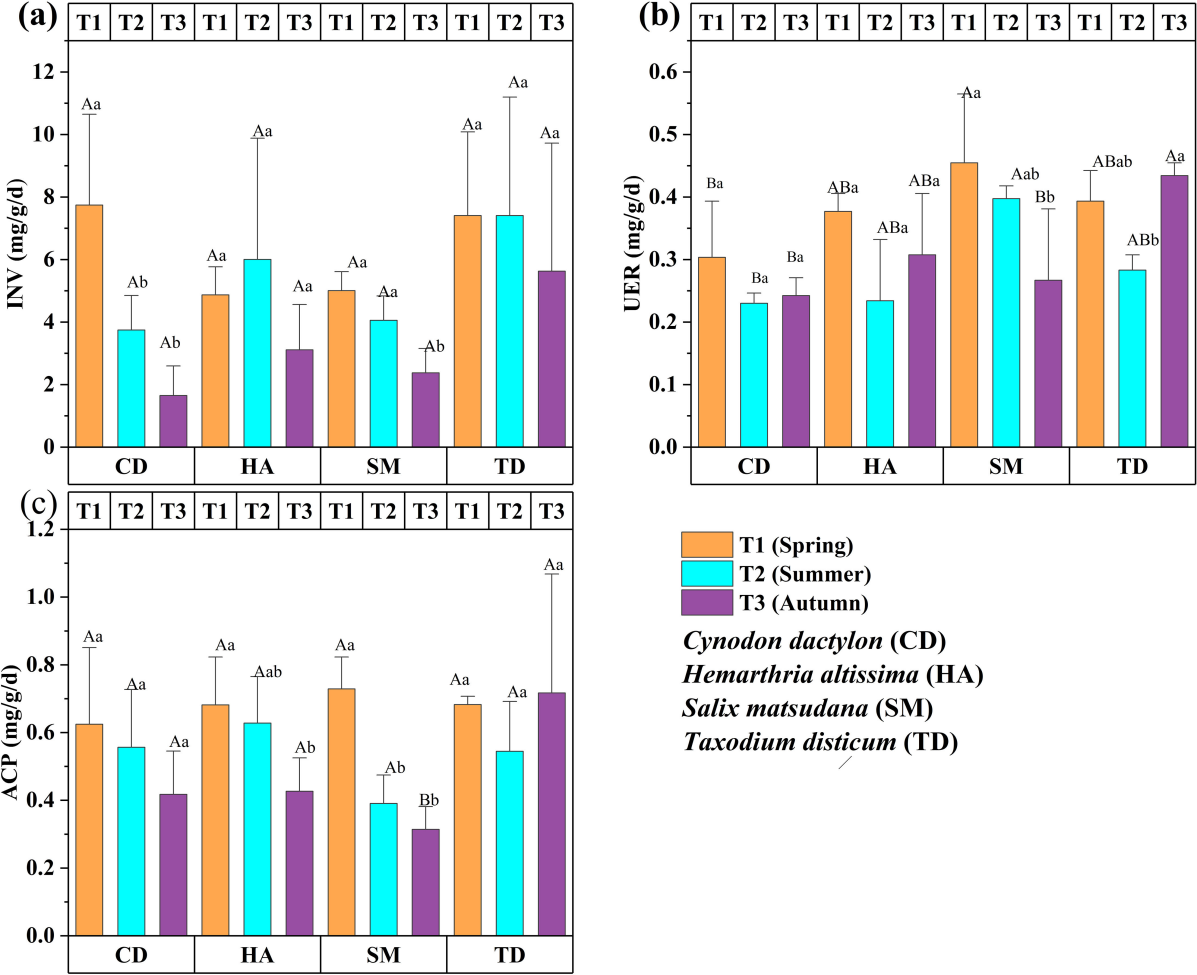
Bar graphs **(a–c)** showing the enzyme activities of four vegetations at different periods in the riparian zones of the TGDR in China. Abbreviations in figures define invertase content (INV), soil urease content (URE), and acid phosphate content (ACP). Uppercase letters indicate significant differences between vegetation types in the same period, while lowercase letters show differences across periods (*p < 0.05*).

Enzymatic activity exhibited significant seasonal and vegetation-specific patterns assessed over three seasons (T1, T2, T3) for CD, HA, SM, and TD. INV was generally highest in T1, with CD and TD showing significantly higher levels in spring (*p < 0.05*), reflecting active carbohydrate metabolism during early growth stages (**Figure 4a**). For SM, URE peaked in T1 (*p < 0.05*), which means nitrogen cycling was better in spring. On the other hand, TD had higher UER activity in T3, which meant that nitrogen metabolism was better in T3 (*p < 0.05*) (**Figure 4b**). ACP was highest in T3 for TD (*p < 0.05*), reflecting enhanced phosphorus mobilization in autumn, whereas SM showed higher ACP activity in T1, declining significantly in T2 and T3 (*p < 0.05*). CD and HA maintained relatively stable ACP levels across seasons (**Figure 4c**).

#### 3.2.4 Relationships between soil properties

**Figure 5:**
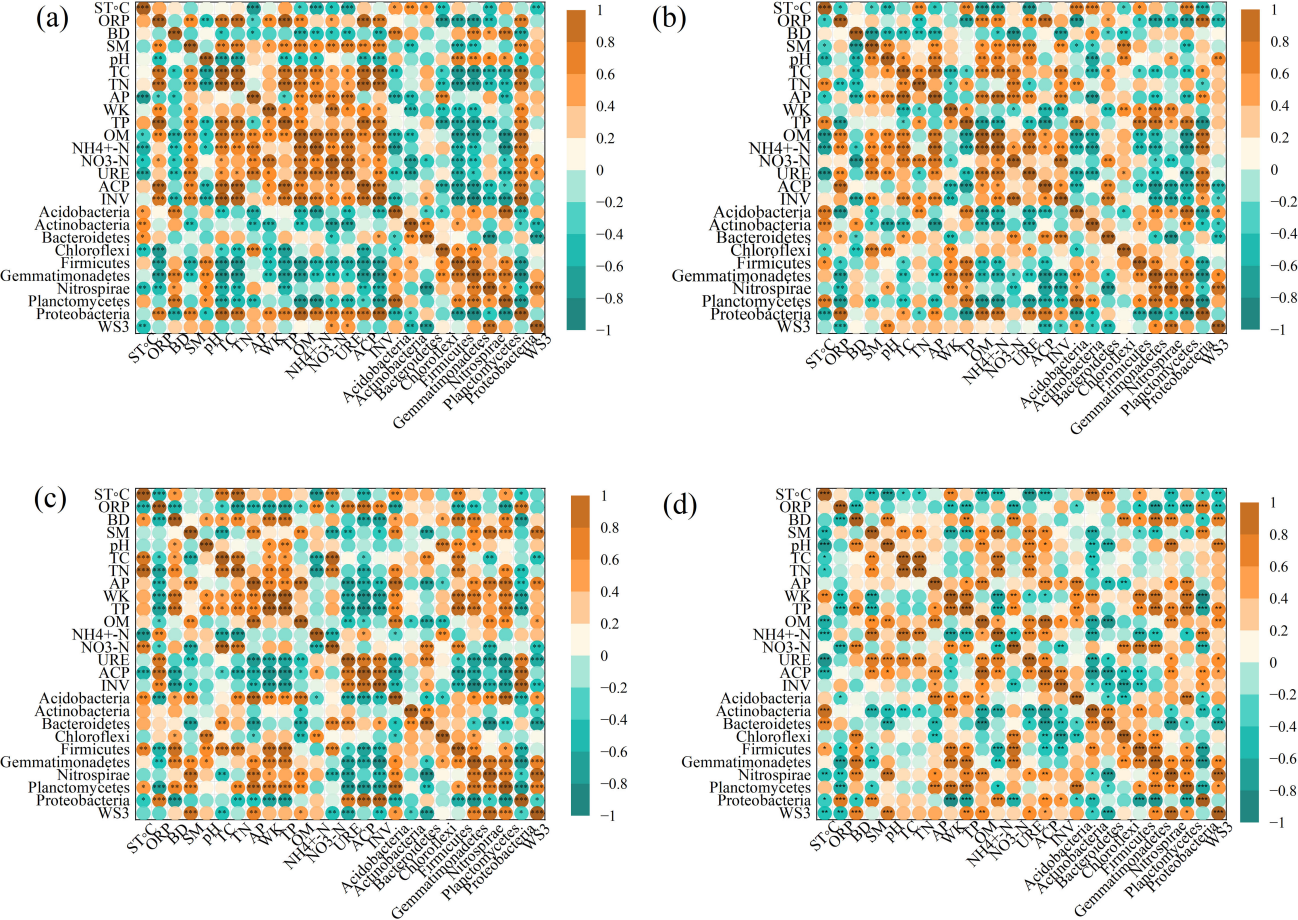
Heat-maps of correlations between soil properties and bacterial communities across seasons. Significant correlations (*p < 0.05*, *p < 0.01*, *p < 0.001*) are shown for CD **(a)**, HA **(b)**, SM **(c)**, and TD **(d)**. Abbreviations in the figure are defined as soil temperature (ST °C), oxidation-reduction potential (ORP), bulk density (BD), soil moisture content (SMC), pH values (pH), total carbon (TC), total nitrogen (TN), available phosphorous (AP), whole potassium (WK), total phosphorous (TP), organic matter (OM), ammonium nitrogen (NH_4_^+^-N), nitrate nitrogen (NO_3_- N), urease (URE), soil phosphatase (PHO), and soil invertase (INV). The symbols represent statistical significance levels: * denotes p < 0.05, ** denotes p < 0.01 , *** denotes p < 0.001.

A heat map analysis (**Figures 5a–d**) also showed strong links (*p < 0.05*) between soil features and bacterial communities in all four types of vegetation (CD, HA, SM, and TD). Soil pH positively correlated with *Nitrospirae* (r = 0.603**) and *Proteobacteria* (r = 0.558**), favoring bacterial groups involved in nitrification. TP correlated with OM (r = 0.785***) and NH₄#x207A;-N (r = 0.661***), highlighting phosphorus’s role in nitrogen availability and organic matter decomposition. AP correlated with *Acidobacteria* (r = 0.721***) and *Actinobacteria* (r = 0.565**), promoting microbial diversity and nutrient cycling. TN correlated with *Firmicutes* (r = 0.622***) and *Chloroflexi* (r = 0.605***), enhancing nitrogen-fixing bacteria. URE correlated with NH₄⁺-N (r = 0.512**) and OM (r = 0.624***), supporting nitrogen cycling under high organic matter. ACP correlated with AP (r = 0.821**), emphasizing enzymatic roles in phosphorus mobilization. These findings demonstrate how soil properties shape microbial communities and nutrient cycling across ecosystems.

#### 3.2.5 Principal component analysis

**Figure 6:**
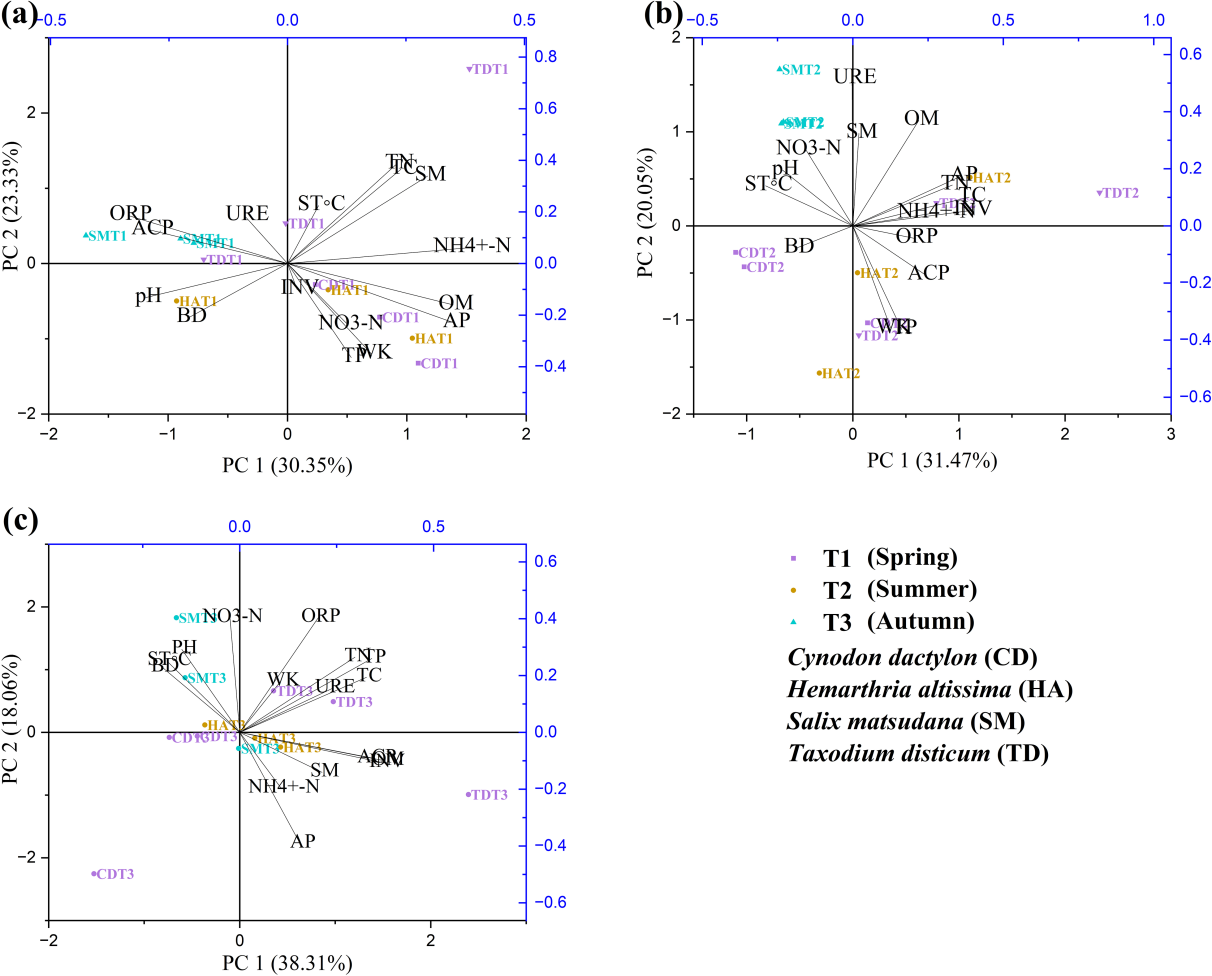
Principal component analysis (PCA) of soil physiochemical properties over different seasons **(a–c)** in riparian zones of the TGDR in China. Abbreviations in the figure are defined as soil temperature (ST°C), oxidation-reduction potential (ORP), bulk density (BD), soil moisture content (SMC), pH values (PH), total carbon (TC), total nitrogen (TN), available phosphorous (AP), whole potassium (WK), total phosphorous (TP), organic matter (OM), ammonium nitrogen (NH_4_^+^-N), nitrate nitrogen (NO_3_-N), urease (URE), soil phosphatase (PHO), and soil invertase (INV).

**Figure 6** highlights the key soil properties identified through PCA assessed over three seasons (T1, T2, T3) for CD, HA, SM, and TD. The variability across spring (T1) was mainly captured by PC1 (30.35% variance) (influenced by NH₄⁺-N, OM, and AP) and PC2 (23.33%) (contributions from TN, TC, and ORP) (**Figure 6a**), demonstrating the importance of nutrient availability and redox dynamics during spring. PC1 (31.47%) in T2 focused on OM, AP, and INV, while PC2 (20.05%) captured SM, UER, and NO₃-N, explaining moisture and enzymatic activity as important summer drivers (**Figure 6b**). At T3, PC1 (38.31% variance) was dominated by OM, TP, and TC (and likely nutrient storage and litter decomposition); PC2 (18.06%) was identifiable as highlighting redox dynamics and nitrate availability (**Figure 6c**). Soil samples were found to cluster distinctly by season and vegetation type, with T1 samples linked to nutrient availability, T2 associated with enzymatic activity and moisture, and T3 related to phosphorus and organic carbon dynamics using PCA biplots.

### 3.3 Microbial community dynamics

#### 3.3.1 Seasonal and vegetation-specific soil microbial community dynamics

**Figure 7:**
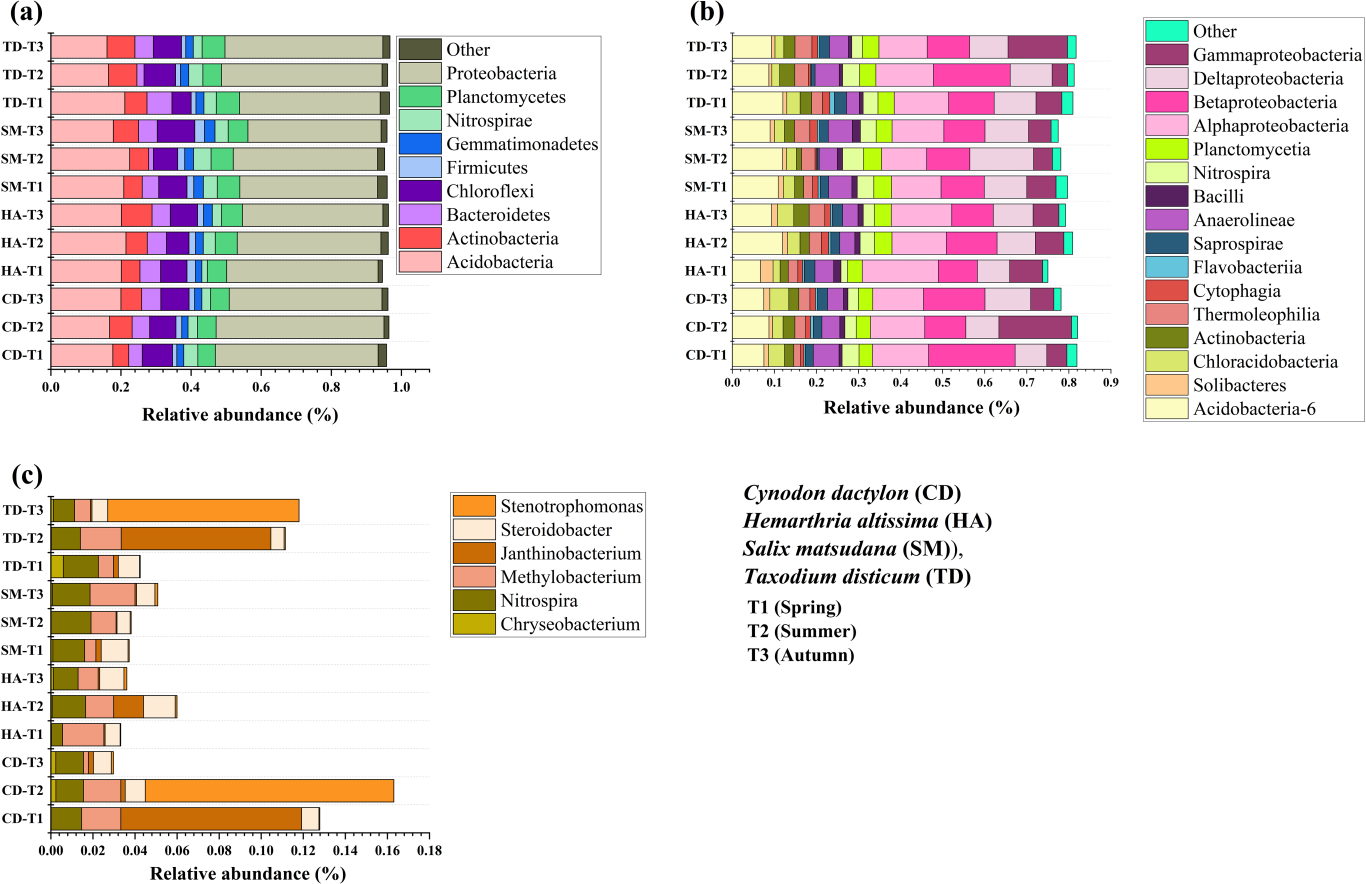
Stacked column plots of the relative abundance of bacterial sequences of four vegetations at different periods in the riparian zones of the TGDR in China at the phylum **(a)**, class **(b)**, and genus **(c)** levels.

A comprehensive analysis of riparian soils identified 68 bacterial phyla and 211 classes, with *Proteobacteria* and *Acidobacteria* dominating, accounting for over 60% of the relative abundance. Seasonal and vegetation-specific variations were assessed over three seasons (T1, T2, T3) for CD, HA, SM, and TD (*p < 0.05*). *Proteobacteria* were most common in TD during T3 and SM during T2. They are linked to the cycling of nutrients and the breakdown of organic matter. *Acidobacteria* were more abundant in CD and HA during T2, emphasizing their adaptability to soil nutrient fluctuations. Other groups, such as *Actinobacteria*, increased during T3 in SM, reflecting their role in litter-fall decomposition (**Figure 7a**). *Alphaproteobacteria* and *Betaproteobacteria* were the most common at class level. They were most common in TD and SM during T2 and T3, which is when nitrogen fixation and carbon cycling happen (**Figure 7b**). *Nitrospira* was the most common in CD and SM during T2, and *Methylobacterium* was most common in HA during T1. These bacteria were important for taking in nutrients, breaking down nitrogen, and cycling carbon, and there were seasonal patterns that were statistically significant (*p < 0.05*) (**Figure 7c**).

#### 3.3.2 Seasonal and vegetation-specific dynamics of alpha diversity

**Figure 8:**
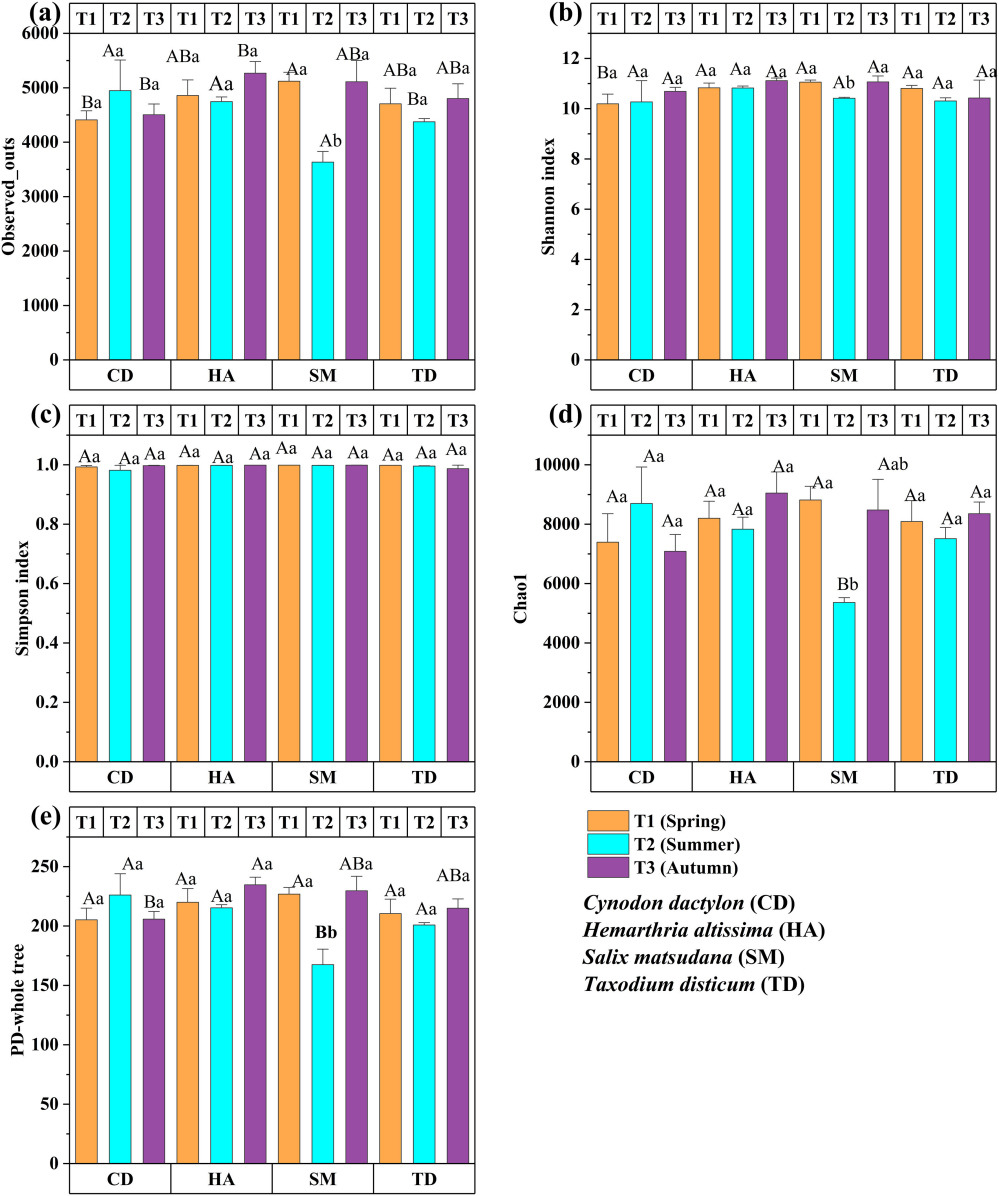
Bar graphs **(a–e)** depict the α-diversity indices of bacterial communities across different seasons in the riparian zones of the TGDR, China. Observed OTUs are shown in **(a)**, the Shannon index in **(b)**, the Simpson index in **(c)**, the Chao1 index in **(d)**, and the PD-whole tree index in **(e)**. Uppercase letters indicate significant differences between vegetation types in the same period, while lowercase letters show differences across periods (*p < 0.05*).

**Figure 8** presents the seasonal changes in soil bacterial diversity across CD, HA, SM, and TD, assessed over three seasons (T1, T2, T3). OTUs and Chao1 indices peaked in T3 for CD, SM, and TD (*p < 0.05*), with HA showing higher values in T2 and T3 (*p < 0.01*) (**Figures 8a, d**). The Shannon index decreased significantly for SM in T2 (*p < 0.01*) (**Figure 8b**) while the Simpson index remained stable (*p > 0.05)* (**Figure 8c**). PD increased significantly in T3 for SM and TD (*p < 0.05*) and CD (*p < 0.01*) (**Figure 8e**). These findings emphasize T3 as a peak period for microbial richness and diversity in riparian zones.

#### 3.3.3 Seasonal and vegetation-specific of β diversity analysis in soil bacterial communities

**Figure 9:**
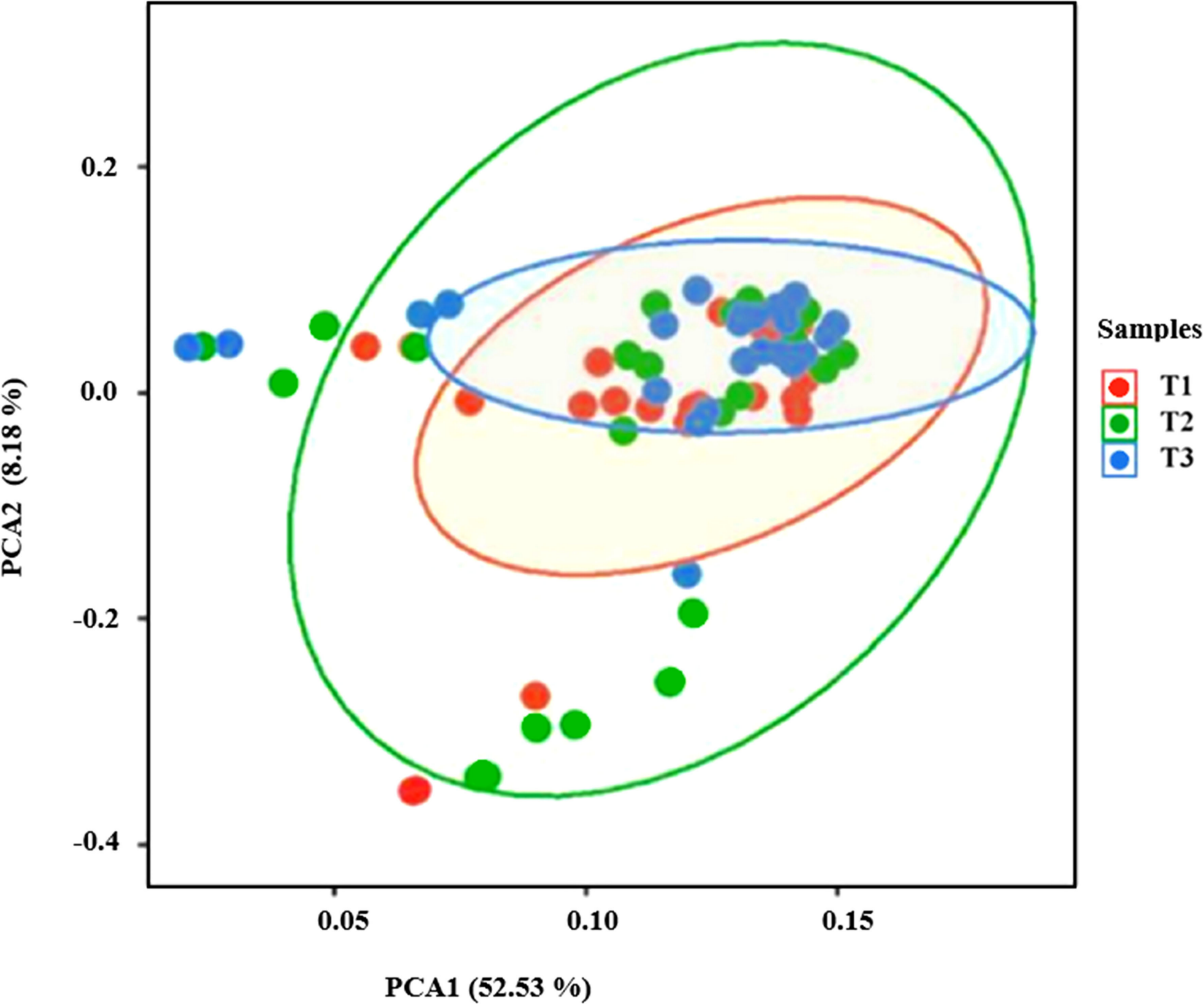
The PCA plot illustrates the variation in soil bacterial community composition across three seasons (T1: Spring, T2: Summer, and T3: Autumn) in the riparian zones of the TGDR. The ellipses represent the 95% confidence intervals for each season, highlighting the seasonal clustering of bacterial communities.

PCA revealed distinct patterns of soil bacterial community β diversity assessed over three seasons (T1, T2, T3). The first two principal components, PCA1 (52.53%) and PCA2 (8.18%), cumulatively explained 60.71% of the variation (**Figure 9**). The PCA plots demonstrated a consistent distribution of soil samples across seasons, with minimal variations in diversity. This suggests stable bacterial community structures with minimal seasonal fluctuations. These seasonal changes in the makeup of the microbial community show how different types of plants, nutrient availability, and the environment all affect each other. This shows how the changing conditions in the soil ecosystem affect the microbes that live there.

### 3.4 Functional and pathway analyses

#### 3.4.1 Seasonal and vegetation-specific functional characterization of soil bacterial communities

**Figure 10:**
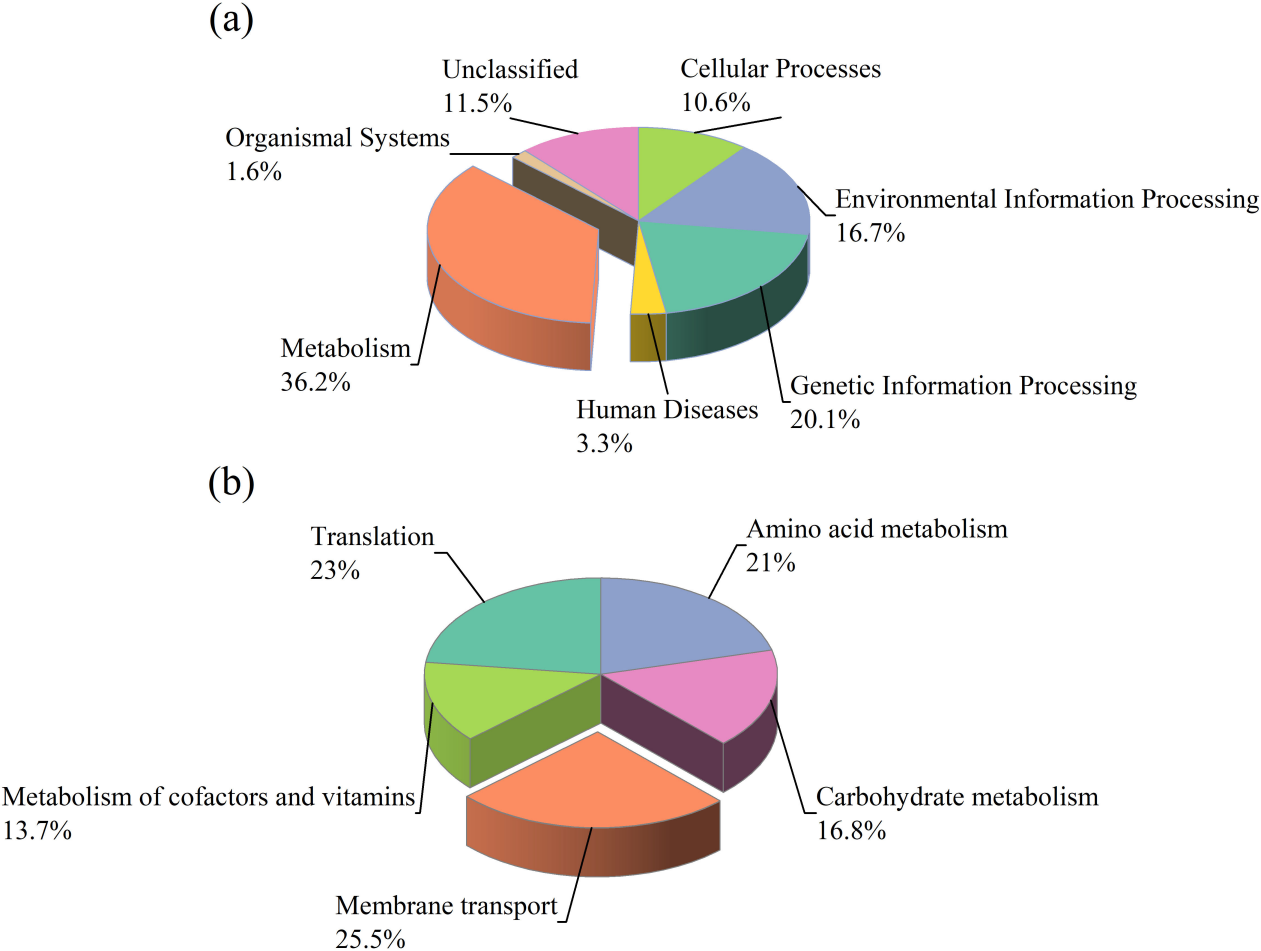
The pie chart **(a)** illustrates the functional distribution of bacterial communities in the riparian zones of the TGDR based on KEGG pathway analysis. The pie chart **(b)** shows the distribution of secondary KEGG pathway functions in bacterial communities from the riparian zones of the TGDR.

We used PICRUSt to perform functional prediction of soil bacterial communities based on the KEGG database for six primary pathways: metabolism (36.2%), genetic information processing (20.1%), environmental information processing (16.7%), cellular processes (10.6%), organismal systems (1.6%), and diseases (3.3%) (**Figure 10a**). This study found that metabolic function predictions made from soil samples with NSTI scores between 0.07 and 0.13 (mean 0.10) were very accurate. These scores matched the reference microbial genome database very well. Latitudinal and vegetation-specific differences also strongly influenced the shifts in functional pathways, with tree species TD and SM exhibiting functional shifts during the T3 due to increased nutrient cycling and organic matter inputs. Functional profiles across seasons were more stable for herbaceous species CD and HA, indicating consistent environmental adaptation. The results show how important the metabolic and genetic information processing pathways are across all of the soil samples. This means that microbial community is very important for keeping the soil ecosystem stable. There were 45 gene pathways linked to six main metabolic groups in the secondary KEGG orthology (KO) functional classification. Membrane transport (25.5%), translation (23%), amino acid metabolism (21%), carbohydrate metabolism (16.8%), and cofactors and vitamins metabolism (13.7%) were the most abundant functions (**Figure 10b**).

#### 3.4.2 Cluster dynamics of soil bacterial communities

**Figure 11:**
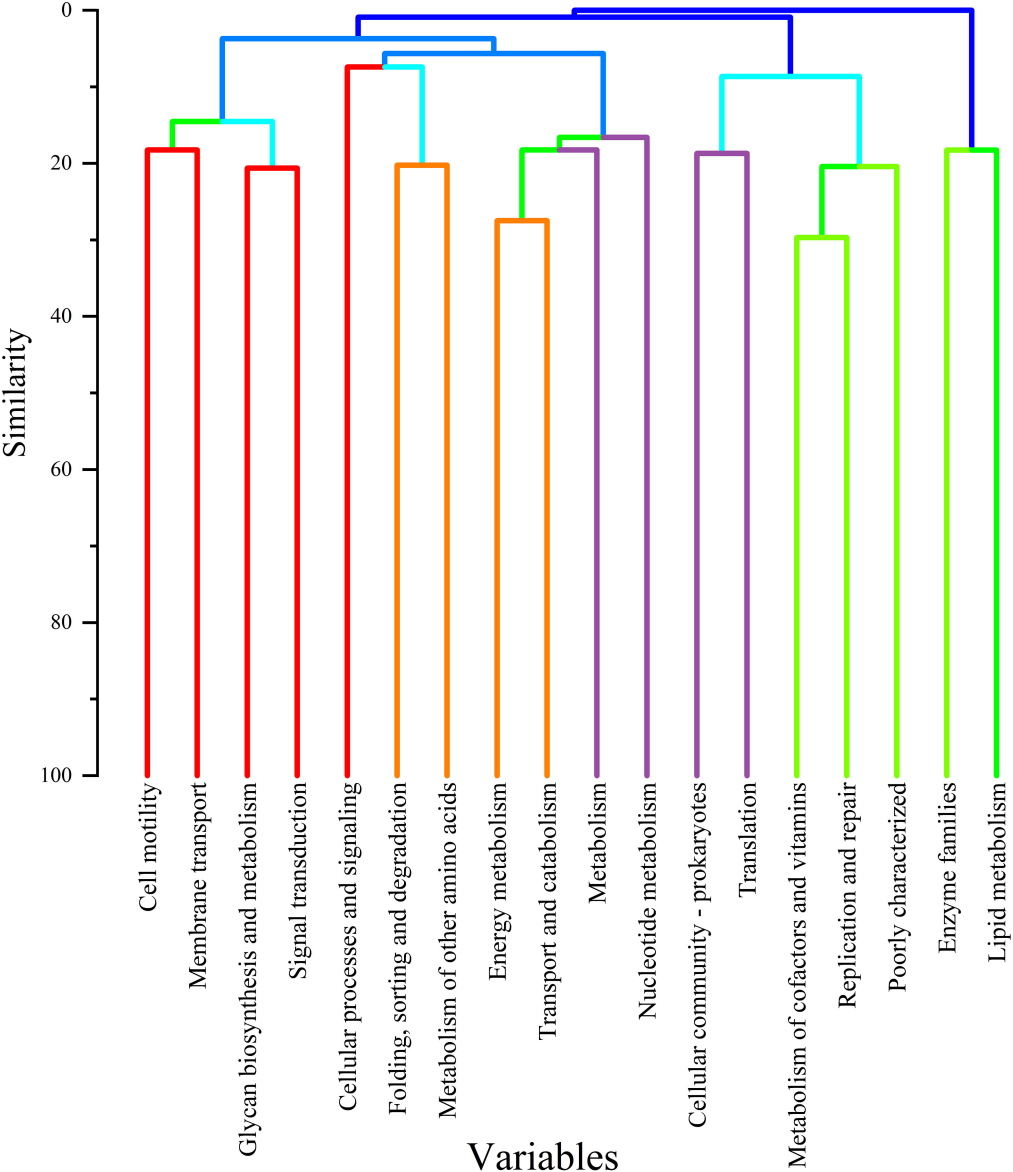
Clustering map of the relative abundance of key metabolic genes at the functional level.

Cluster analysis revealed that TD and HA exhibited similar microbial responses during T3, as illustrated in the hierarchical clustering dendrogram (**Figure 11**), indicating heightened metabolic activity and nutrient cycling. The dendrogram highlighted distinct functional clusters, with lipid metabolism and enzyme families forming the closest group (80–90% similarity), followed by cofactor/vitamin metabolism and translation (70–80%). Other clusters included nucleotide metabolism (60–70%) and transport/catabolism (50–60%), while specialized functions like energy metabolism and membrane transport were more distinct (40–50%). This clustering underscores the synchronized microbial activity in TD and HA, driven by seasonal changes, supporting ecosystem stability and plant-microbe interactions during T3.

## 4 Discussion

### 4.1 Influence of ARPs on soil properties, microbial diversity, and ecosystem functions

This study revealed the significant impact of ARPs on soil properties, microbial diversity, and ecosystem functions within the riparian zones of the TGDR. Specifically, tree-dominated ARP species, including TD and SM, demonstrated substantial improvements in soil structure, organic matter content, and nutrient retention. The T3 period, characterized by peak litterfall, amplified these effects by providing large inputs of organic material that drive nutrient cycling and microbial activity. Under these systems, soil stability increased due to increased enzymatic activity of ACP and URE, which are implicated in phosphorus and nitrogen cycling. In this study, we discovered that organic matter accumulation also contributes to soil stability. This work aligns with previous studies that affirm the crucial role of soil enhancement in the dynamic riparian zone (Yu et al., 2023; Zhang D. et al., 2020). ARPs supported resilient microbial communities with dominant taxa, such as *Proteobacteria* and *Acidobacteria*, that adapted quickly to vegetation-specific inputs. Functional pathways like carbohydrate metabolism, amino acid metabolism, and lipid metabolism were activated in vegetation-dominated ARPs (TD and SM) in the autumn, showing that they were active with microbes and moving nutrients around. In this context, these pathways serve as a means for the microbial community to adapt to organic inputs, particularly litter decomposition. This promotes ecosystem functions during seasonal peaks. They contribute greatly to the stabilization of soil ecosystem functions, according to Kim et al. (2021) and Yang et al. (2023). The positive feedback loop between vegetation inputs, microbial activity, and soil nutrient availability makes ARPs ecologically relevant. This finding fits with what Ye et al. (2015) and Zhang et al. (2021) found: that organic inputs from plants help microbes divide and keep ecosystems working in riparian systems.

Moreover, tree-dominated ARP species contributed to increased microbial diversity and supported organic matter decomposition and nutrient cycling. Second, seasonal variations further corroborate the effectiveness of these systems in response to seasonal fluctuations in environmental parameters, particularly enzymatic activities. Our observations align with those of Xu et al. (2023) and Zhao et al. (2020), who also noted similar seasonal microbial adaptation in riparian ecosystems with significant hydrological variability. ARPs are also a suitable strategy, especially with adaptive species such as TD and SM, for improving soil properties, microbial diversity, and ecosystem functions in the TGDR. Dam-regulated riparian zones effectively guard against environmental degradation and foster long-term ecological recovery (Xiao et al., 2021; Wei et al., 2019). The evidence demonstrated the global relevance of ARPs as a sustainable riparian restoration approach in dynamic environmental and anthropogenic pressure contexts. It also underscored their role in safeguarding large river biodiversity.

### 4.2 Seasonal changes and ARPs effectiveness

ARPs in the riparian zones of the TGDR were highly sensitive to seasonal variability. In all cases, T3 was the most effective season for ARPs, coinciding with increased litter decomposition, higher enzymatic activity, and peaks in microbial diversity. The richness and evenness of the microbial community were apparent from oscillations in the Shannon and Chao1 alpha diversity indices. These indices showed significant seasonal fluctuations. Seasonal changes in the functional pathways of microbes that have been observed, such as higher carbohydrate metabolism in CD and SM during T2 and higher amino acid metabolism in TD and HA during T3, are in line with the availability of seasonal organic matter, since litter breaks down mostly in the fall. However, these functional shifts represent how ARPs, particularly tree species such as TD, respond to seasonal changes and greatly increase microbial activities at critical nutrient cycling times. PCA analysis reinforced this seasonal clustering by revealing different soil properties and microbial functionality patterns. Tree species such as TD were particularly effective during wet periods, as they supplemented high organic inputs and litterfall, leading to higher nutrient mobilization and microbial activity. These findings corroborate previous studies by Conradie and Jacobs (2020) and Lin et al. (2023), highlighting the importance of hydrological and climatic variation for structuring riparian soil ecosystems. In more arid seasons, CD and HA maintained soil stability and microbial functionality. They had extensive root systems that reduced soil erosion, conserved water, and retained microbes in water-limited conditions. This approach demonstrates the seasonal adaptive capacity of ARPs. This reflects plant traits, environmental condition dynamics, and answers the second research question by suggesting that increasing ARPs will enhance ecosystem resilience to seasonal extremes. These results highlight the importance of incorporating seasonal adaptive strategies into ARP application designs to gain the greatest ecological benefit. The responses make it possible for the ARPs to adapt quickly to changes in the seasons. This keeps the soil stable all year and promotes nutrient cycling and microbial health, which are in line with the main goals for long-term riparian restoration in environments that are always changing.

### 4.3 Most effective plant species for ARPs

TD outperforms other selected species in improving soil and microbial properties, particularly during autumn. These species significantly boost the activity of crucial enzymes such as ACP and URE, essential for phosphorus and nitrogen movement in the soil. TD also increased the variety and abundance of microbes, which helped with the cycling of nutrients and the breakdown of organic matter (He et al., 2021; Lyu et al., 2023). T3 also increased the activity of functional networks associated with TD, such as lipid metabolism and amino acid metabolism. These shifts are particularly significant for nutrient cycling and organic matter decomposition, and they align with peak litter inputs. The consistent ability to support such a functional shift further characterizes TD as one of the most suitable species for seasonally adaptive ARPs when organic feed availability is variable. Furthermore, SM demonstrated high versatility, enabling it to provide organic matter and root exudates to improve the microbial community. Under wet conditions, TD effectively complements these processes, maintaining microbial activity and nutrient cycling. However, CD and HA, which are herbaceous species, thrive during the dry seasons due to their capacity to retain soil and water through their extensive root systems, thereby maintaining their stability. These grass-stabilized riparian species demonstrated greater resilience to drought than control species, particularly when faced with water-limiting conditions. Our findings are consistent with accounting for locally adapted and species-specific strategies for seasonally adapted ARPs, as outlined in Jiajia et al. (2023). ARP initiatives may help maximize riparian restoration in the TGDR and other similar habitats around the world through the combination of strengths among tree and grass species.

### 4.4 Comparison with existing research

Seasonal and vegetation-specific variations in soil enzymatic activity and nutrient cycling observed in this study are consistent with prior research. Results by Jiang et al. (2023) and Li et al. (2022b) reinforce the role of seasonality in controlling soil microbial respiration and enzyme activity. This study further shows that organic matter inputs, especially during autumn, also affect soil processes. The finding of *Proteobacteria* and *Acidobacteria* dominance in this study agrees well with a study by Kim et al. (2021) and suggests their adaptation to changing environmental conditions. Zhang et al. (2022) demonstrated that riparian zone water level fluctuations may impact nitrogen cycling, a conclusion supported by our findings. The study of how microbes move nitrogen and phosphorus around supports Daims et al. (2015) found about *Nitrospira*’s significant role as a nitrifier and what Li et al. (2022c) found about how nutrient imbalances change microbes’ activity and carbon storage. These studies emphasize the ecological importance of nitrogen-cycling genes in response to seasonal nutrient fluctuations. This study also shows that tree-dominated species, like TD, have changing responses to soil. This fits with Zhao et al. (2023) and Shen et al. (2022) findings about how vegetation and water levels affect enzyme activity and nutrient cycling. This study enhances our understanding of the role of vegetation in riparian systems. The work of Farrell et al. (2020) and Bhatti et al. (2017a, b) adds to what Tang et al. (2021) found about how flooding and replanting affect nitrogen dynamics. This shows how imperative it is to include microbial dynamics in ecosystem restoration efforts. Our findings connect microbial pathways to broader ecosystem processes and help link land management strategies to sustainable management and riparian ecosystem functioning.

### 4.5 Interpretation and ecological implications

Our integrative analyses reveal a multifaceted picture of soil ecosystem dynamics. We observed strong positive correlations between SOM, TC, TN, pH, AP, and TP with key microbial enzyme activities, including URE and ACP. Conversely, BD demonstrated a negative correlation with SOM and TC, highlighting its limited effect on nutrient availability. These findings emphasize that seasonal dynamics profoundly shape soil functionality. Tree species such as TD and SM exhibited significant seasonal shifts—especially during the T3 period—when increased leaf litter and organic matter decomposition drive enhanced nutrient cycling and enzyme activity. In contrast, herbaceous species like CD and HA maintained relatively stable conditions, indicating a consistent contribution to soil structure throughout the seasons. The PCA analysis further clarified these patterns by linking T1 to nutrient availability, T2 to moisture and enzymatic activity, and T3 to nutrient storage and decomposition. Given the significant increases in enzymatic activity and nutrient mobilization during autumn, our results support the idea that riparian zones function as nutrient and microbial hotspots during periods of peak organic matter input. The activation of metabolic pathways—including carbohydrate, amino acid, and lipid metabolism—underscores the microbial community’s adaptive response to seasonal organic inputs, stabilizing nutrient cycling and enhancing ecosystem resilience. These insights reinforce the ecological significance of ARPs in riparian restoration. They suggest that the selection of vegetation, particularly tree species that promote dynamic nutrient cycling, is critical for sustaining long-term soil functionality and ecological stability in environments subject to hydrological variability.

### 4.6 Theoretical and practical contributions

These results support the resource availability hypothesis that some soils are rich in seasonally available resources. This may contribute to high soil microbial diversity and enzymatic activity. SM and TD show strong proof that these microbes use functional pathways in very different ways at different times of the year. This strongly supports the idea that seasonal resources play a key role in shaping microbial communities and ecosystems. The results reveal alterations in crucial pathways like carbohydrate metabolism and lipid metabolism. This indicates the direct impact of vegetation inputs on nutrient cycling and microbial activity. This suggests the potential role of functional pathways in advancing riparian ecosystem management strategies in the future. Earlier research on seasonal peaks in microbial diversity and enzymatic function, except for T3, supports the idea that resource supply is important to the structure of the soil microbial ecosystem (Babur et al., 2021; Rebello et al., 2018). Ren et al. (2015) and Zhong et al. (2018) show that vegetation contributes to nutrient cycling stabilization and overall ecosystem functionality in the ever-changing riparian zone. These results provide immediate practical implications for sustainable land management strategies. This study highlights the ecological benefits of vegetation-specific adaptations, such as the nutrient cycling efficiency of tree species, and proposes actionable approaches for riparian ecosystem management (Meng et al., 2023). Previous research on vegetation-driven ecosystem services includes vegetation that would increase retention of nutrients, soil health, and conservation of biodiversity (Dosskey et al., 2010; Marapara et al., 2020). These insights lay the groundwork for optimizing ecosystem services in riparian landscapes. They also comprehend the impacts of environmental variability and manage land use pressures.

### 4.7 Limitations and future directions

Despite its limitations, this study offers insightful perspectives on seasonal and vegetation-specific processes in riparian soil ecosystems. The three seasonal sampling points allow us to observe broader patterns but may not capture more fine temporal variations in microbial dynamics or enzymatic activity. Also, KEGG pathway-based functional predictions are helpful, but they are based on inferred data. Added direct metagenomic validation could make predicted microbial functions more accurate. Addressing these limitations and expanding the scope of future research to investigate the effects of more extreme climatic events, such as droughts or floods, could provide important insights into microbial resilience and adaptation. Microbial diversity and functionality across timescales in relation to environmental change are best studied over extended periods. Additionally, studies of rare microbial taxa may indicate their potential role in biogeochemical cycling of nutrients or ecosystem stability. This research illustrates how complex interactions of the riparian soil ecosystem can be created by vegetation, seasonal inputs, and microbe enzymatic events. It highlights the significance of plant-specific adaptations and the seasonality of nutrient cycling, enzyme kinetics, and microbial community structure in the management and restoration of these valued ecosystems.

## 5 Conclusions

This study examined the effectiveness of ARPs in changing soil properties, microbial diversity, and ecosystem functioning in the riparian zones of the TGDR. We found that TD and SM positively impacted nutrient cycling and microbial diversity. In addition, other ARPs enhanced the physical, chemical, and enzymatic properties of the soil. We found that microbial activity was more seasonal than previously recognized and peaked with organic matter inputs from vegetation during autumn. We performed functional pathway analysis and showed that microbes could adapt by increasing amino acid and nitrogen cycling when nutrients were plentiful. There is more and more recognition that interactions between plants and microbes help keep ecosystems stable, especially in big systems controlled by dams like the TGDR. This study identifies key species, TD and SM, as critical for ARPs, offering practical guidance for ecological restoration. The temporal scale of this study and the absence of long-term monitoring pose limitations. Future research could investigate the effects of other functional traits, scrutinize finer temporal scales, and examine ARP effectiveness under extreme climatic events. This research could be extended to other riparian systems to assess ARP performance under a range of contrasting environmental pressures. Overall, this study contributes to ecological restoration science and provides useful ecological implications for restoring sustainable riparian systems amid climate change and anthropogenic impacts.

## Data Availability

The datasets presented in this study can be found in online repositories. The names of the repository/repositories and accession number(s) can be found in the article/**Supplementary Material**.
