# Supplementary material for: Bacterial communities and soil functionality in artificially remediated vegetation of the three gorges reservoir zone
Source: Front Plant Sci. 2025 Apr 28;16:1550306. doi: 10.3389/fpls.2025.1550306 (PMC12066796; doi:10.3389/fpls.2025.1550306)
Supplement: Supplementary file 1 [file Table1.docx]

Supplementary Material

## Supplementary Figures


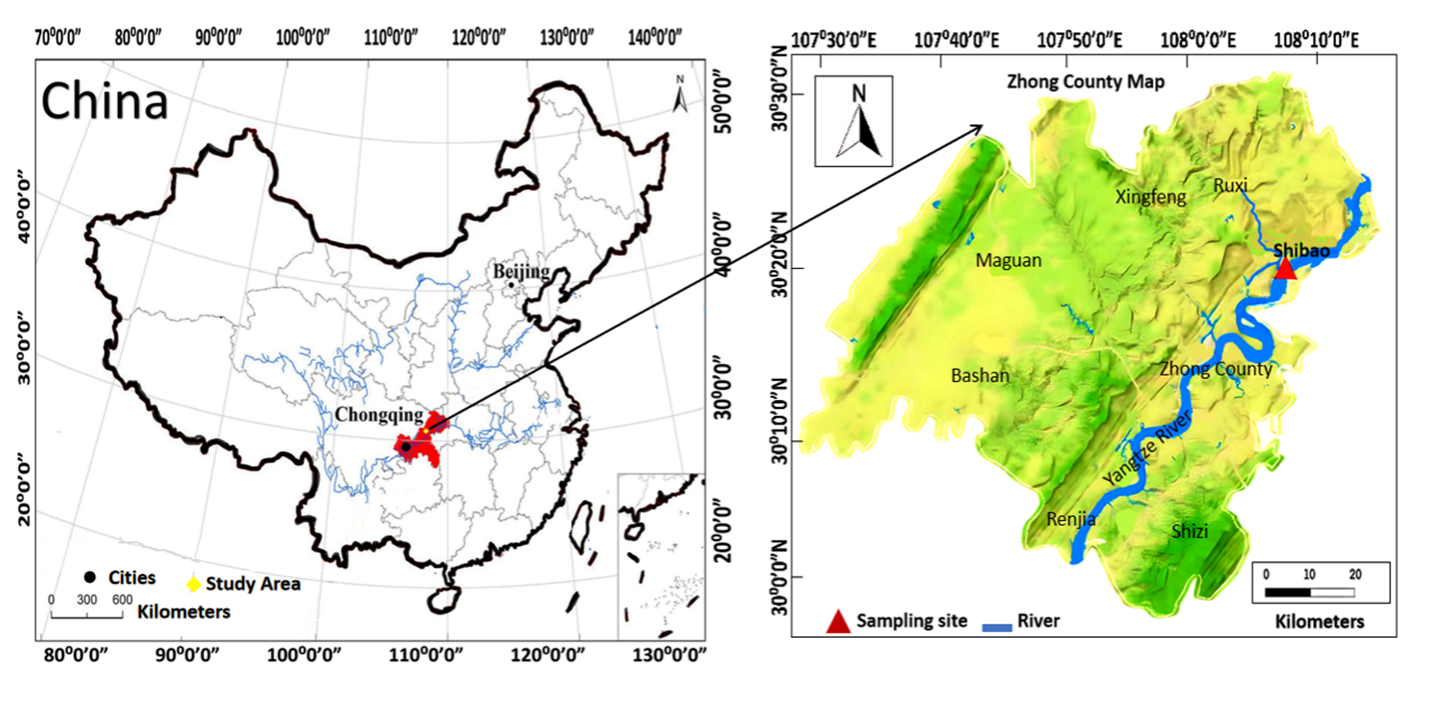


**Supplementary Figure 1.** The geographical map of the study area highlights the riparian zones of the Three Gorges Dam Reservoir (TGDR). It features the sampling sites marked with red triangles, located in Zhong County along the Yangtze River within the Chongqing Municipality.


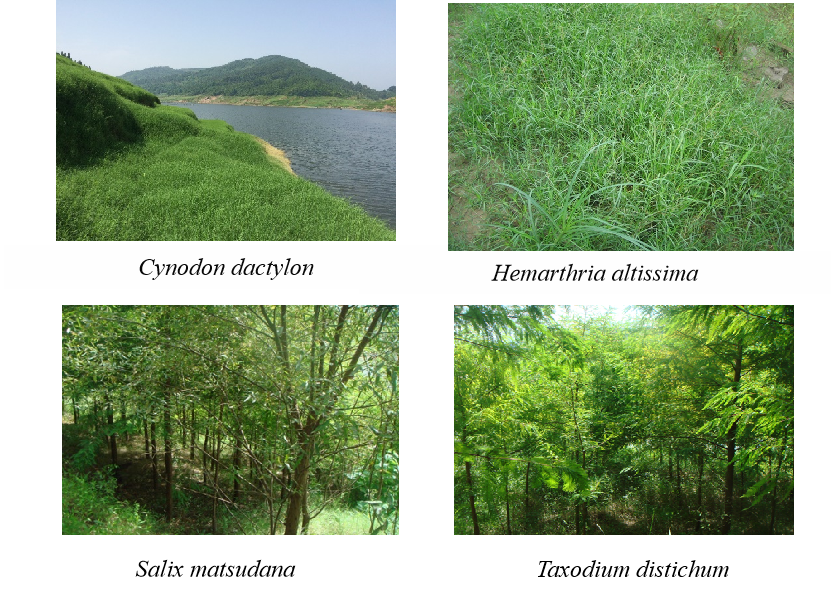


**Supplementary Figure 2.** Photographs of the sampled plant species from the riparian zones of the Three Gorges Dam Reservoir in China. These images showcase the vegetation studied, which plays a significant role in the ecological dynamics of the area.
